# Endobronchial Malignant Melanoma: An Exceedingly Rare Occurrence

**DOI:** 10.7759/cureus.8619

**Published:** 2020-06-14

**Authors:** Marium Ghani, Sara R Skavroneck, Kiran Adlakha, Alfred Papali

**Affiliations:** 1 Internal Medicine, Wyckoff Heights Medical Center, New York, USA; 2 Internal Medicine, Carolinas Medical Center - Atrium Health, Charlotte, USA; 3 Pathology, Carolinas Medical Center - Atrium Health, Charlotte, USA

**Keywords:** melanoma, metastasis, endobronchial, pulmonary, lung

## Abstract

Malignant melanoma is rarely observed to metastasize to endobronchial tissue. We present a case of endobronchial malignant melanoma in a 36-year-old male smoker with a regressed cutaneous lesion. Due to the limited number of cases and poor survival rate, no definitive treatment options are available to improve survival in patients with this rare disease presentation. Immunotherapy and surgical removal of locally aggressive tumor have been described, but the definitive role for these therapeutic modalities in the setting of endobronchial metastases remains largely unknown.

## Introduction

Extrapulmonary primary tumors commonly metastasize to the lung parenchyma, but rarely to the airways. As of 2004, only 204 cases of endobronchial metastases had been described in the English literature over 40 years [1]. Malignant melanoma comprises 4.5% of all endobronchial metastases arising from extra-thoracic primary sites [2]. We report a case of endobronchial malignant melanoma in a 36-year-old male smoker with a regressed cutaneous lesion.

## Case presentation

**Figure 1:**
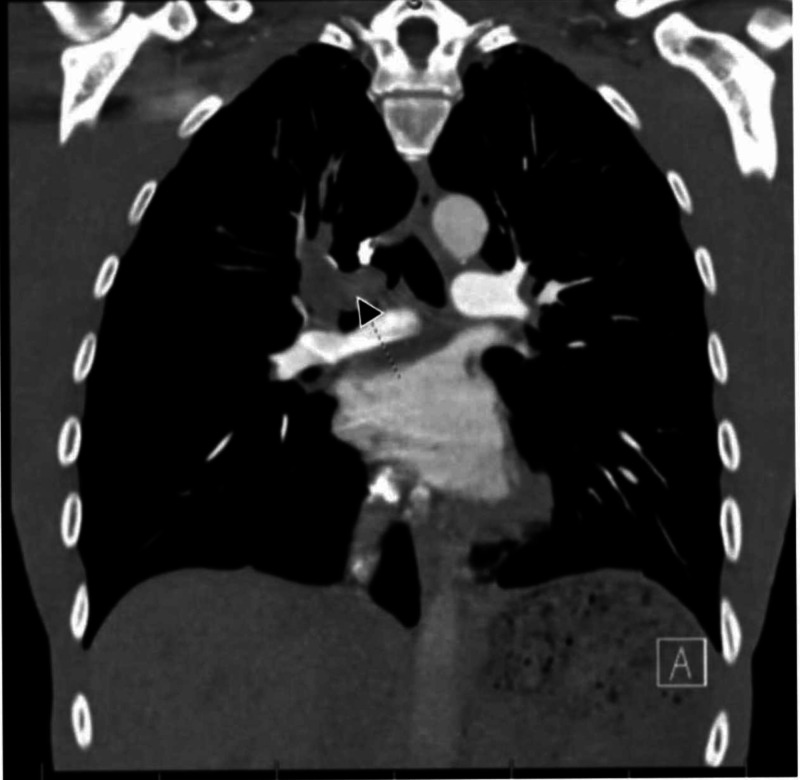
Large right upper lobe endobronchial mass

A 36-year-old Caucasian man presented with acute hemoptysis, worsening cough, dyspnea, and chest pain. He had a 20 pack-year smoking history and worked in landscaping with significant sun exposure. Computed tomography (CT) angiography of the chest revealed a right upper lobe endobronchial mass effacing the distal right mainstem bronchus (Figure 1).

**Figure 2:**
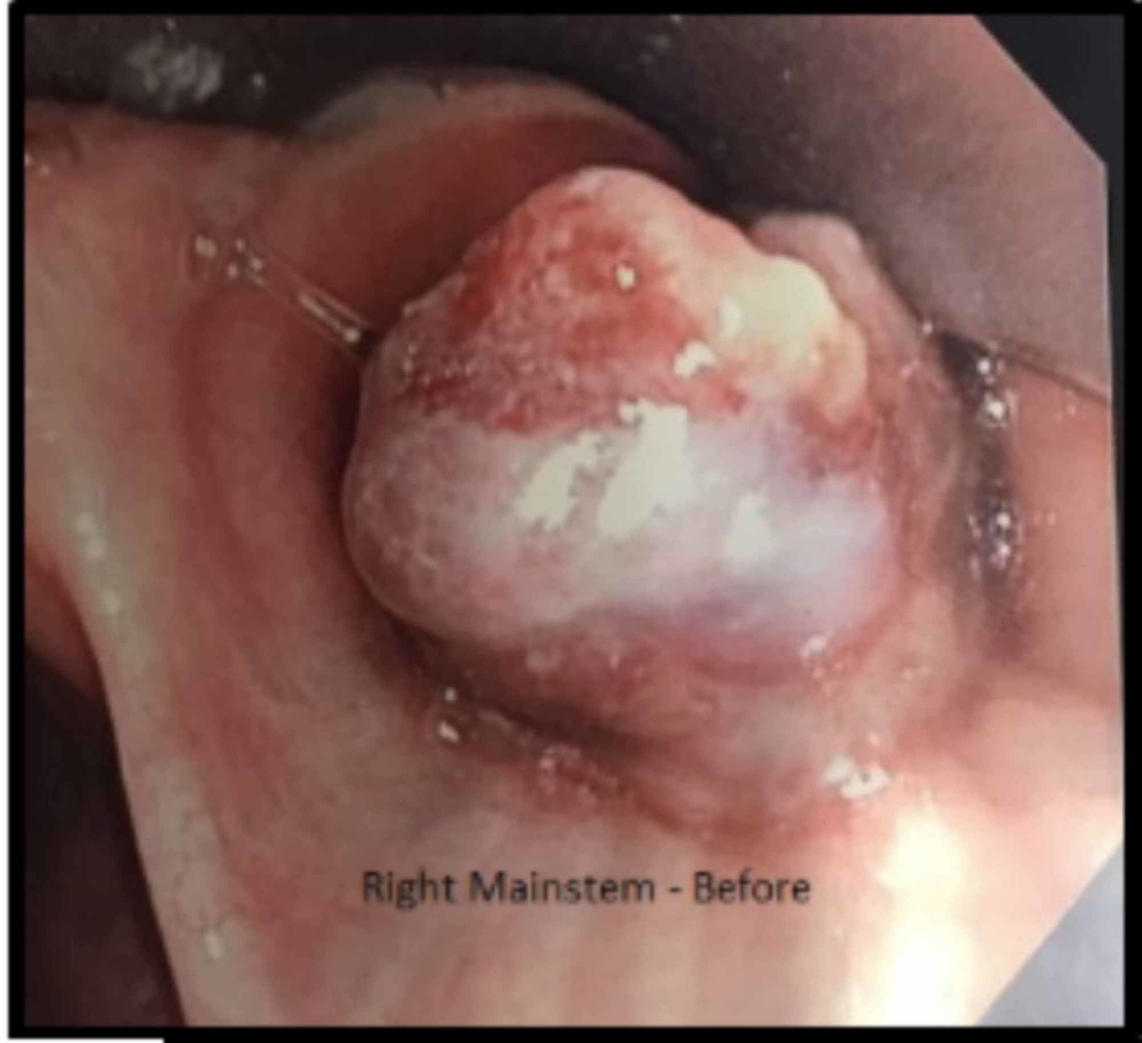
Carina with near-complete obstruction of the right mainstem bronchus

Flexible bronchoscopy revealed a large, friable endobronchial mass with near-complete obstruction of the proximal right mainstem bronchus (Figure 2).

**Figure 3:**
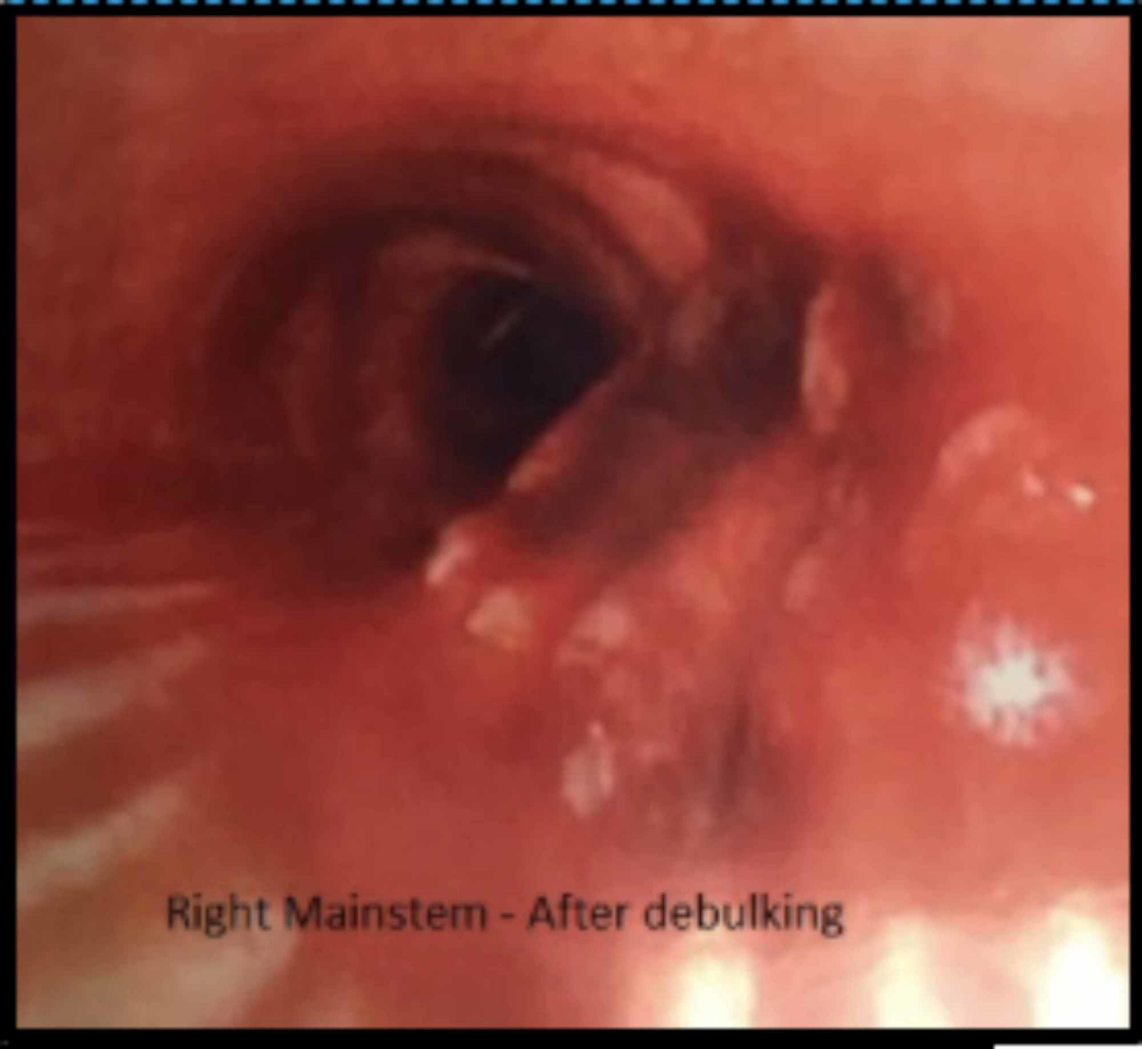
Right mainstem bronchus after debulking

The tumor was successfully debulked with rigid bronchoscopy using a hexagonal snare, argon plasma coagulation, and raptor forceps (Figure 3).

**Figure 4:**
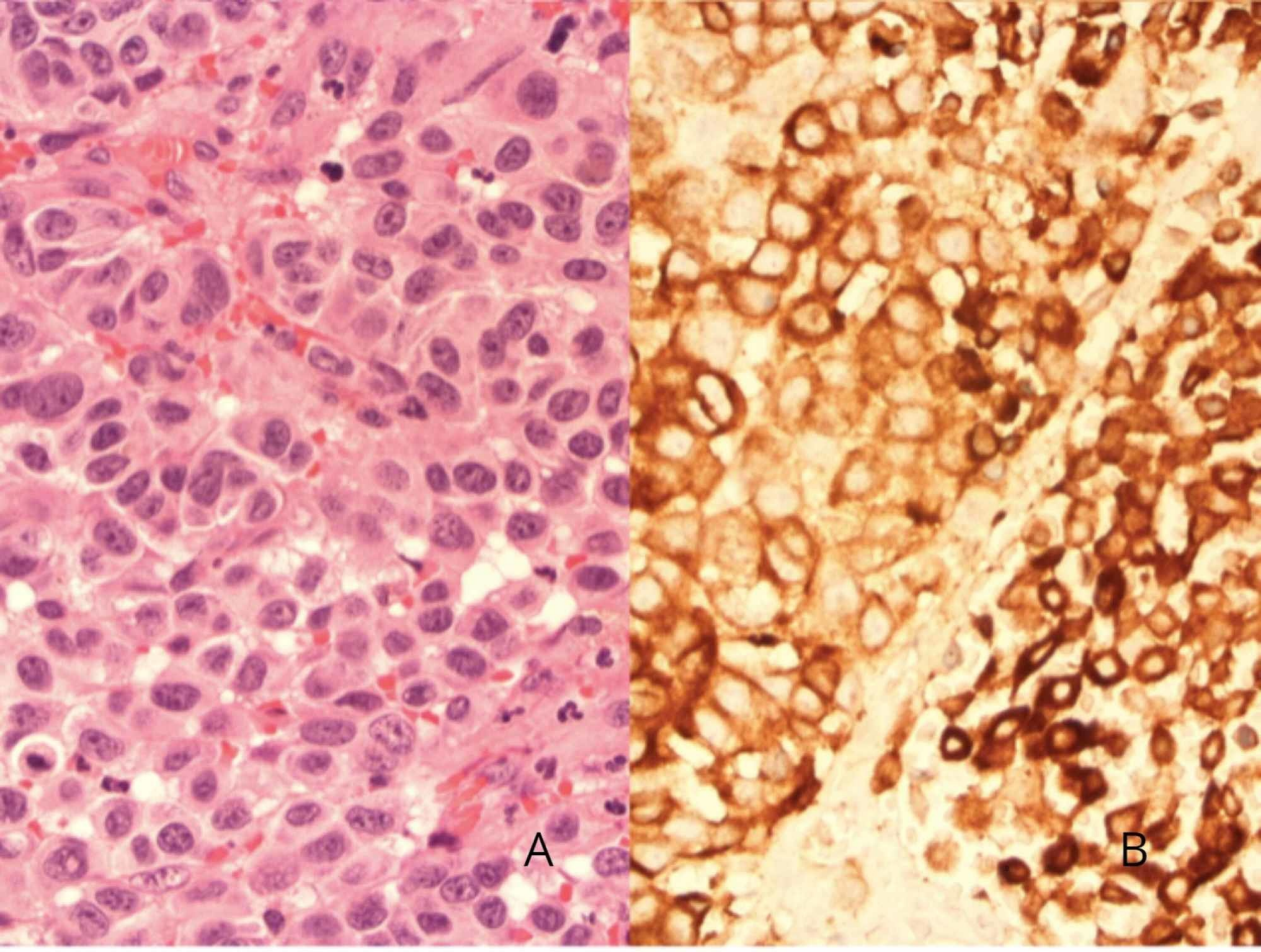
(A) Hematoxylin and eosin (H&E) stain in high power; (B) Positive MART-1 immunostaining of the endobronchial mass MART-1: melanoma antigen recognized by T cells

Symptoms improved significantly post-operatively. Surgical biopsy of the specimen demonstrated malignant cells, which on immunohistochemistry stained positive for S100 (marker of Schwann cells and melanocytes) and melanoma antigen recognized by T cells (MART-1) and negative for p40, cytokeratin 7 (CK7), and thyroid transcription factor-1 (TTF-1) (Figure 4).

These findings in conjunction with histologic features helped in rendering the diagnosis of malignant melanoma. Subsequent history revealed that he had “picked off” a pruritic, bleeding mole that had been increasing in size over the prior three to six months. Comprehensive dermatologic inspection revealed a central back nevus with evidence of regression. Positron emission tomography (PET) scan showed bilateral hypermetabolic axillary uptake in addition to the known endobronchial focus of disease. The patient declined treatment for advanced malignant melanoma and entered hospice six months later. 

## Discussion

Pulmonary metastases are commonly observed in cases with solid extrapulmonary malignancies; however, an endobronchial invasion is rarely seen. The estimated incidence of endobronchial metastases may vary from 2%-28% [3], of them, only 4.5% (204 cases) were reported due to malignant melanoma. The differential diagnosis for endobronchial malignant melanoma includes primary pulmonary melanoma (PPM) and endobronchial metastasis. PPM typically extends first to the hilar, then to distal lymph nodes. Our patient’s history of a regressing skin lesion and lack of thoracic lymphadenopathy is highly suggestive of endobronchial metastasis, but PPM remains possible since skin involvement was not confirmed by biopsy. The prognosis for endobronchial malignant melanoma remains quite dismal with a median overall survival of six months [3].

Due to the rarity of the disease, there are no published guidelines for the treatment of endobronchial malignant melanoma. Endobronchial resection can be offered as a palliative treatment option for patients with respiratory symptoms, but resection is not curative in the setting of metastatic disease. Dacarbazine was initially the only chemotherapeutic agent approved for metastatic melanoma, but now immunotherapy is the mainstay of treatment [4,5]. Combination therapy nivolumab (a programmed death 1 [PD-1] checkpoint inhibitor) and ipilimumab (a cytotoxic T-lymphocyte-associated antigen 4 [CTLA-4] checkpoint inhibitor) have shown complementary effect and have significantly longer progression-free survival than monotherapy [4,6]. For patients with BRAF gene mutation, which is associated with 36%-45% of primary and 42%-55% of metastatic melanomas [7], vemurafenib, a kinase inhibitor, can be offered [8,9].

(Abstract: Skavroneck S, Adlakha K, Zgoda M, Jacob S, Papali A. Endobronchial Malignant Melanoma: An Exceedingly Rare Occurrence. American Thoracic Society International Conference; May 17-21, 2019). https://cslide-us.ctimeetingtech.com/ats2019_eposter/attendee/eposter/poster/7856?s=pbn

## Conclusions

Our case of endobronchial malignant melanoma highlights the limited treatment options available for patients presenting with this rare disease manifestation. Prognosis for endobronchial melanoma remains extremely poor. Evidence supporting effective treatments are limited. Likewise, the lack of a standardized approach to diagnosis limits early detection. Future clinical studies will be helpful to guide the treatment of this aggressive form of pulmonary malignancy.
